# Simultaneous harvesting of radiative cooling and solar heating for transverse thermoelectric generation

**DOI:** 10.1080/14686996.2021.1920820

**Published:** 2021-06-29

**Authors:** Satoshi Ishii, Asuka Miura, Tadaaki Nagao, Ken-ichi Uchida

**Affiliations:** a International Center for Materials Nanoarchitectonics (MANA), National Institute for Materials Science (NIMS), Tsukuba, Japan; b Faculty of Pure and Applied Physics, University of Tsukuba, Tsukuba, Japan; c PRESTO, Japan Science and Technology Agency, Saitama, Japan; d Research Center for Magnetic and Spintronic Materials, National Institute for Materials Science (NIMS), Tsukuba, Japan; e Department of Condensed Matter Physics, Graduate School of Science, Hokkaido University, Sapporo, Japan; f Institute for Materials Research, Tohoku University, Sendai, Japan; g Center for Spintronics Research Network, Tohoku University, Sendai, Japan

**Keywords:** Spin Seebeck effect, radiative cooling, solar heat, thermoelectric effect, energy harvesting, 40 Optical, magnetic and electronic device materials, 203 Magnetics / Spintronics / Superconductors, 210 Thermoelectronics / Thermal transport / insulators, 204 Optics / Optical applications, 206 Energy conversion / transport / storage / recovery

## Abstract

For any thermoelectric effects to be achieved, a thermoelectric material must have hot and cold sides. Typically, the hot side can be easily obtained by excess heat. However, the passive cooling method is often limited to convective heat transfer to the surroundings. Since thermoelectric voltage is proportional to the temperature difference between the hot and cold sides, efficient passive cooling to increase the temperature gradient is of critical importance. Here, we report simultaneous harvesting of radiative cooling at the top and solar heating at the bottom to enhance the temperature gradient for a transverse thermoelectric effect which generates thermoelectric voltage perpendicular to the temperature gradient. We demonstrate this concept by using the spin Seebeck effect and confirm that the spin Seebeck device shows the highest thermoelectric voltage when both radiative cooling and solar heating are utilized. Furthermore, the device generates thermoelectric voltage even at night through radiative cooling which enables continuous energy harvesting throughout a day. Planar geometry and scalable fabrication process are advantageous for energy harvesting applications.

## Graphical abstract

**Figure:**
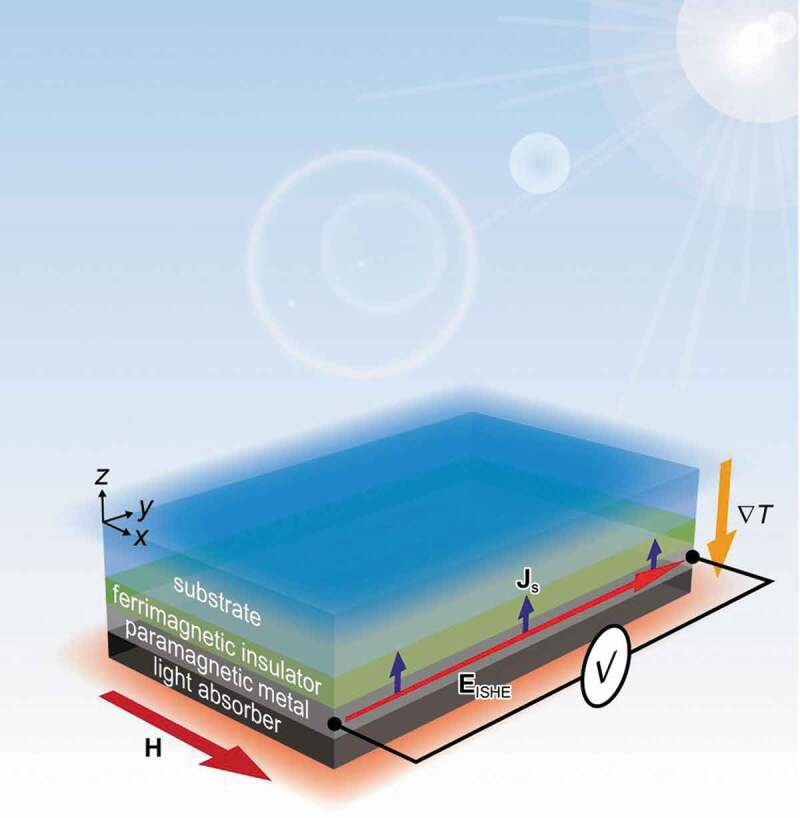

## 1. Introduction

The majority of thermoelectric generation is based on the Seebeck effect, where the temperature gradient and the generated electric field are parallel to one another. Thus, the Seebeck effect is often referred to as the longitudinal thermoelectric effect. In contrast, the transverse thermoelectric effect is another type in which the temperature gradient and the thermoelectrically generated electric field are perpendicular to each other. The spin Seebeck effect (SSE) is one particular form of the transverse thermoelectric effect [1–4]. In an SSE device, the SSE-induced voltage is proportional to the length of the device which is perpendicular to the temperature gradient. This means that the voltage (power) due to the SSE can increase by simply elongating the device length (enlarging the device area) perpendicular to the temperature gradient without forming multitude of serial p-n junctions as is the case with conventional thermoelectric devices based on the Seebeck effect [5]. The advantages of SSE devices are that they can have a low thickness whilst maintaining a low thermal resistance [6] and their fabrication process and facile scalability [7].

When an SSE device has a low thickness, the temperatures at the top and bottom of the device easily converges, which results in a decreased thermoelectric voltage. Thus, it is highly desirable to develop a passive method to maintain a distinct temperature gradient which can simultaneously cool one side and heat the other. For the heating sources, in addition to waste heat, photothermal heating [8–10] including solar heat can be utilized. In particular, solar heat has been considered as a heat source in thermoelectric modules [11–13], some of which include hybrid systems with photovoltaic cells [14,15]. In contrast to the heat sources, passive cooling sources are often not readily available. One possible solution to this problem is by utilizing outdoor radiative cooling. Radiative cooling generally occurs when an object is placed outside at night with a clear sky which causes the object to thermally radiate in the mid-infrared (MIR) spectrum to the extremely cold space. Previous studies have investigated generating thermoelectric power by radiative cooling at night [16,17]. Although radiative cooling is dominated by solar heating in the daytime, it is possible to achieve daytime radiative cooling by utilizing advanced surface designs [18–20]. A daytime radiative cooling structure reflects or scatters incoming sunlight without absorption and thermally radiates to the space in the MIR [21–23]. Sub-ambient cooling in daytime has been experimentally achieved by utilizing numerous designs and materials [24–32]. However, previous designs did not aim to harvest solar heat because sunlight was wasted either by reflection or scattering.

In the current investigation, we prepared a device which is able to simultaneously harvest radiative cooling and solar heating in the daytime to generate transverse thermoelectric voltage by the SSE. This can be achieved with a sunlight-transparent and thermally emissive substrate at the top and sunlight absorbing layer at the bottom while having the SSE structure in the middle. The design is somewhat similar to a daytime radiative cooling structure which has separated thermal radiation and sunlight reflecting layers. However, in the prepared device, a sunlight absorbing layer was utilized instead of a sunlight reflecting layer at the bottom. For the proof-of-concept demonstration, we used a hybrid device consisting of a paramagnetic gadolinium gallium garnet (GGG)/ferrimagnetic yttrium iron garnet (YIG)/paramagnetic platinum (Pt) trilayer and blackbody (BB) paint where the GGG/YIG/Pt trilayer is a prototypical structure for the SSE. Critically, the GGG side was facing upwards during the measurements where the GGG and Pt/BB layers were the thermal radiation and sunlight absorbing layers, respectively. The outdoor measurements were verified by indoor measurements, which imitated outdoor conditions, and by numerical heat transfer simulations. Our investigation advances the practical applications of SSE devices, which are planar and scalable, to be used in self-power off-grid sensors within the era of Internet of Things (IoT).

## 2. Device concept

**Figure 1.**
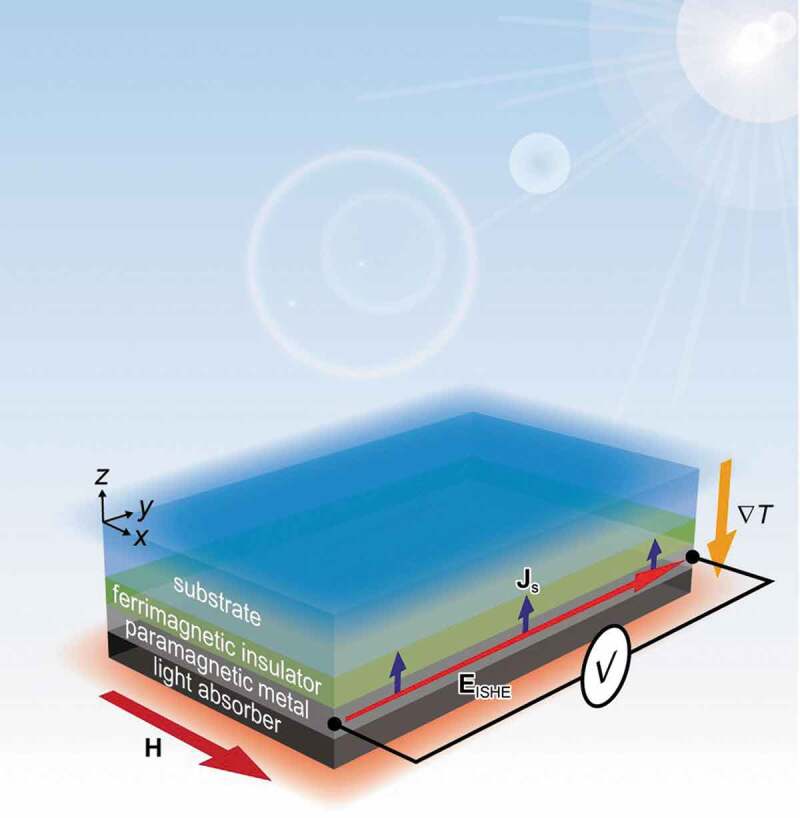
Schematic of the device which generates thermopower based on the SSE by radiative cooling at the top substrate and solar heating at the bottom light absorber. The image depicts the situation where the sample is placed outdoor on a sunny day. A temperature gradient, magnetic field vector, spatial direction of the spin current generated by the SSE, and electric field generated by the ISHE are respectively expressed as Δ*T*, **H, J**_s_, and **E**_ISHE._

Figure 1 depicts a schematic of the prepared device which can simultaneously harvest radiative cooling and solar heating to generate thermoelectric voltage by the SSE. The device consists of four layers in order; a substrate, a ferrimagnetic (or ferromagnetic) insulator, a paramagnetic metal, and a light absorber. High transparency in the solar spectrum and high emissivity in the atmospheric window are the necessary conditions for the substrate. Additionally, the ferrimagnetic insulator has to be transparent to sunlight in order not to be heated by the sun. The ferrimagnetic insulator and paramagnetic metal form a spintronic device to generate a spin current by the SSE where ferrimagnetic insulator (paramagnetic metal) acts as a heat-to-spin (spin-to-charge) current converter [7]. The paramagnetic metal converts the SSE-induced spin current to electric voltage by the inverse spin Hall effect (ISHE) [33–35]. The purpose of the light absorber is to absorb sunlight which is not fully absorbed by the paramagnetic metal.

When the device is placed outside on a sunny day, the substrate emits thermal radiation to the universe and gets cooler because it is transparent in the solar spectrum and emissive in the MIR [10,36]. Simultaneously, the incoming sunlight is mostly absorbed by the paramagnetic metal and light absorber to heat the bottom side. This is because the substrate and ferrimagnetic insulator are transparent in the solar spectrum. Thus, both radiative cooling at the top side and solar heating at the bottom side generate a negative temperature gradient along the *z*-axis (i.e. temperature gradient toward the negative *z*-direction). This temperature gradient excites magnons in the ferrimagnetic insulator and induces a spin current in the paramagnetic metal. By applying an external magnetic field along the *y*-axis, the spin current is converted to electric voltage by the ISHE to be recorded as *V*_ISHE_ (see Section 3.3 and Figure S2 where the procedures for extracting the pure *V*_ISHE_ contribution are shown). In principle, the external magnetic field is not required to obtain *V*_ISHE_ if the remanent magnetization of the ferrimagnetic insulator is finite.

In the current work, a prototypical device was realized by using GGG, YIG, Pt, and BB as the substrate, ferrimagnetic insulator, paramagnetic metal, and light absorber, respectively. Since the transparency of YIG is not high in the visible, the YIG thickness was set to be 2 μm which was sufficient to excite magnons for the SSE [7], while being thin enough to be semi-transparent in the visible range. The Pt film thickness (5 nm) was comparable to the spin diffusion length of Pt, and therefore the film was effective in converting the SSE-induced spin current to electric voltage. In the experiment, all the voltage data presented in this paper were obtained from the same sample, allowing us to quantitatively investigate the weather dependence of *V*_ISHE_.

## 3. Experimental section

### 3.1. Sample preparation

A GGG substrate had 2 μm-thick YIG layers grown on both of its sides by liquid-phase epitaxy and was then cut into 7.5 × 7.7 mm^2^ sections. The YIG layer on one side was completely removed by mechanical polishing and the YIG layer on the other side was used for measuring the SSE. After the polishing, the thickness of the GGG/YIG was 0.42 mm. The YIG/Pt junction was prepared in the same manner as in the previous study [7] where a 5 nm-thick Pt film was deposited on the well-polished YIG surface by the radio-frequency magnetron-sputtering. Two electrical contacts were attached by indium near the ends of the Pt film in the *y*-direction. The Pt surface was then painted by a BB spray (JSC-3, Japan Sensor Co.). The total thickness of the GGG/YIG/Pt/BB device was 0.43 mm.

### 3.2. Optical characterization

The reflectance (*R*) and transmittance (*T*) spectra of the device were measured from the GGG side and BB side with UV-visible (V-570, JASCO Co., Japan) and Fourier transform infrared spectrometers (Nicolet iS50R FT-IR, Thermo Fisher Scientific K.K., USA). The absorptance (*A*) was obtained as *A* = 100*−T−R*. In order to evaluate the absorption of each layer, numerical electromagnetic simulations in two dimension were performed using a commercial software based on the finite element method (COMSOL Multiphysics). In the simulations, the complex permittivities of GGG, YIG, and Pt were taken from the references [10,37] and that of the BB was extracted from its reflectance spectrum. Each layer thickness was set based on the actual sample. Scattering boundary conditions and perfect electric conductors were used as the boundary conditions parallel and perpendicular to the samples, respectively.

### 3.3. Measurement of V_ISHE_ induced by the SSE

For both indoor and outdoor measurements, the procedure described in this section was adopted to measure the *V*_ISHE_ originating from the SSE. The sample was placed in the middle of an electromagnet with ~1.5-mm wide support and a magnetic field was applied along the *x*-direction. Cold sink nor thermal grease was not used. The magnetic field was swept from −10 to 10 mT, and the voltage was measured by a nanovolt meter (Keithley 2182A, Tektronix, USA). The voltage measurements at each condition were repeated three times with the GGG side upward and then the BB side upward. At each magnetic-field-dependent measurement, the measured voltage in our Pt/YIG system saturated when the magnitude of the field was larger than 5 mT. As such, *V*_ISHE_ was defined by taking one half of the difference between the saturated voltages at the positive and negative magnetic fields. When the temperature gradient was pointing toward the negative *z*-direction, the sign of *V*_ISHE_ was defined to be positive.

### 3.4. Outdoor measurements

The device was placed together with the electromagnet on the roof of a five-floor building at the National Institute for Materials Science in Tsukuba, Japan (36°04ʹ07.8”N, 140°07ʹ58.7”E). There was nothing that thermally insulated the sample setup from the surrounding. Repeating three measurements at each condition took approximately 4 min. The measurements were carried out on 4th, 5th, 14th, and 16 May 2019.

### 3.5. Indoor measurements

To imitate outdoor radiative cooling, a Peltier module (LVPU-40, VICS Co. Ltd., Japan) was placed above the sample facing downward. A 100 × 100 mm^2^ aluminium plate was coated by the BB paint and attached to the cooling surface of the Peltier module to ensure high emissivity. The distance between the sample and the Peltier module was ~25 mm. The sizes of the device and aluminium plates, in addition to the distance separating them were carefully considered to ensure that the view factor of radiative heat transfer was near unity. During the measurements which involved radiative cooling, the aluminium plate was cooled down to 0°C. A solar simulator (PEC-L01, Peccell Technologies, Inc., Japan) was used as artificial sunlight to irradiate the sample at the incident angle of ~45°. Normal irradiance was not possible because the Peltier module blocked the normal direction. The irradiance at the sample surface was 50 mW/cm^2^. The measurements were performed at room temperature (25°C).

## 4. Results

### 4.1. Optical properties

**Figure 2.**
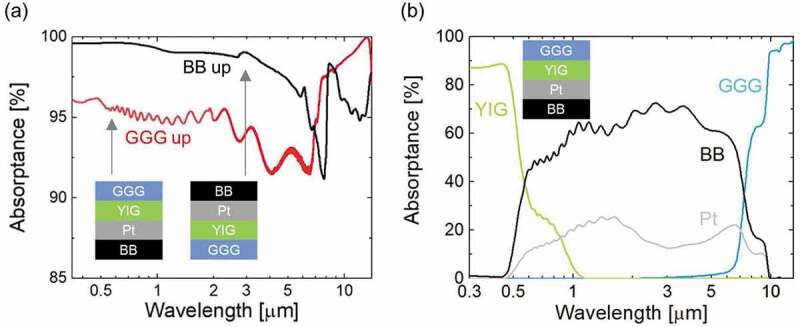
(a) Absorptance spectra in which the measurements were performed from the GGG side (GGG up) and the BB side (BB up). (b) Calculated contribution of each layer to the total absorptance when light is illuminated from the GGG side. The main layers that are contributing to the absorption at *λ* < ~0.5 μm, ~0.5 μm < *λ* < ~8 μm, and ~8 μm < *λ* are YIG, BBB, and GGG, respectively, where *λ* represents wavelength. Note that the sum of the calculated absorptance spectra of four layers has a little discrepancy to the measured absorptance spectrum

Figure 2(a) shows the measured absorptance of the device measured from the GGG side and BB side. Note that the device without the BB layer was semi-transparent in the solar spectrum as shown in Figure S1 because of the very low thickness of the Pt layer. Due to the properties of the BB layer, the measured absorptance of the BB side is high from the UV to MIR range. The measured absorptance from the GGG side is also high in the MIR, although this is not due to the BB layer. To understand the absorptance of each layer when the device was illuminated from the GGG side, the absorptance of each layer was calculated and shown in Figure 2(b). Figure 2(b) elucidates that the main contribution of the absorptance in the MIR, particularly in the atmospheric window, is caused by the GGG. Whereas, the absorptance of the Pt and BB layers dominate in the visible and near IR range. The YIG has a major contributtion in the total absorptance where the wavelength is shorter than ~0.5 μm. Oscillatory features were observed in the absorptance curve for the GGG side in Figure 2(a), in addition to the curves for the Pt and BB layers in Figure 2(b). These are attributed to the interference of the 2 μm-thick YIG layer.

### 4.2. Outdoor experiments

**Figure 3.**
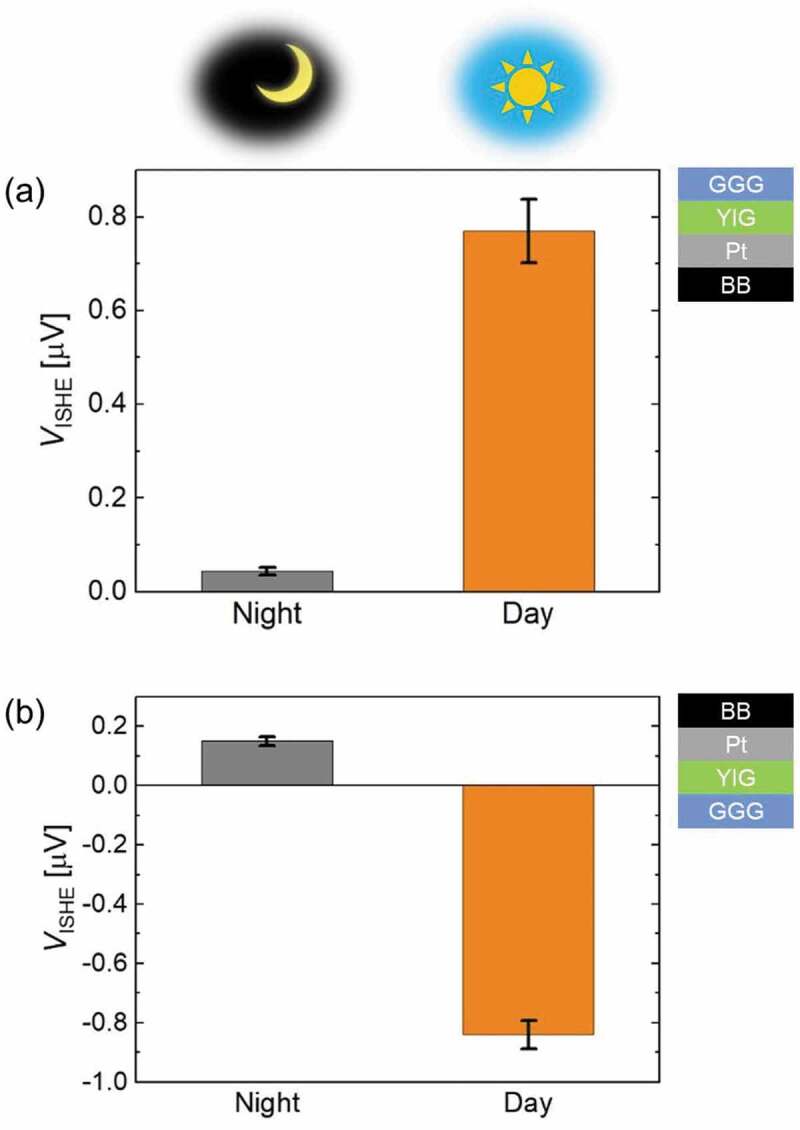
ISHE voltage (*V*_ISHE_) of the device measured outdoors at night and daytime by placing the GGG side upward (a) and the BB side upward (b). The standard deviations of the three measurements that swept the magnetic field at each time are represented by the bars. Sample schematics on the right show the sample orientations during the measurements

The outdoor experiments were performed to investigate the *V*_ISHE_ generation due to radiative cooling and solar heating. Figure 3(a) and (b) present the measured *V*_ISHE_ values when both the GGG and BB sides of the device were facing upward in the daytime and at night (both with a clear sky), respectively. The weather conditions are summarized in Table S1. When the GGG side was facing upward, the *V*_ISHE_ values in day (*V*_ISHE_(day)) and night (*V*_ISHE_(night)) are positive, and *V*_ISHE_(day) is larger than *V*_ISHE_(night). In contrast, when the BB side was facing upward, the signs of *V*_ISHE_(day) and *V*_ISHE_(night) are negative and positive, respectively, and |*V*_ISHE_(day)| is larger than |*V*_ISHE_(night)|.

The observed results can be understood as follows. At night, radiative cooling mainly causes the temperature gradient parallel to the *z*-axis, assuming that convective heat transfer is negligible. High emissivities of the GGG and BB layers in the MIR are expected due to the high absorptances shown in Figure 2(a). As such, the top side of the device was cooled regardless of which side was facing upward to generate a negative temperature gradient. Conversely, when the GGG side was facing upward in daytime, the GGG layer was radiatively cooled while the Pt and BB layers were heated by sunlight. This heating effect increased the negative temperature gradient induced by the radiative cooling of the GGG layer, which in turn increased the *V*_ISHE_ value. However, when the BB side was facing upward in daytime, the BB layer was radiatively cooled and solar heated simultaneously, and the thermal contribution from the other layers of the device was negligible. Since the heat flux of the radiative cooling was lower than the irradiance of sunlight in our setup, as a whole the temperature gradient was generally positive.

The *V*_ISHE_ values recorded on cloudy days are presented in Table S1. On a cloudy day, radiative cooling was reduced because thermal radiation was blocked by the cloud and cannot reach the universe. Nevertheless, the cloud temperature was much lower than the Earth’s surface, such that radiative cooling still occurred albeit with weaker heat flux compared to a clear day. Additionally, solar heating was also weakened due to the cloud coverage. Consequently, these two factors due to cloud coverage decreased the *V*_ISHE_(day) values of the device when the GGG sides was facing upward compared to the respective |*V*_ISHE_(day)| values on a clear day. Similarly, the |*V*_ISHE_(day)| of the sample facing the BB side upward decreased in comparison to a clear day due to the weaker solar heating.

### 4.3. Indoor experiments and heat transfer analysis

**Figure 4.**
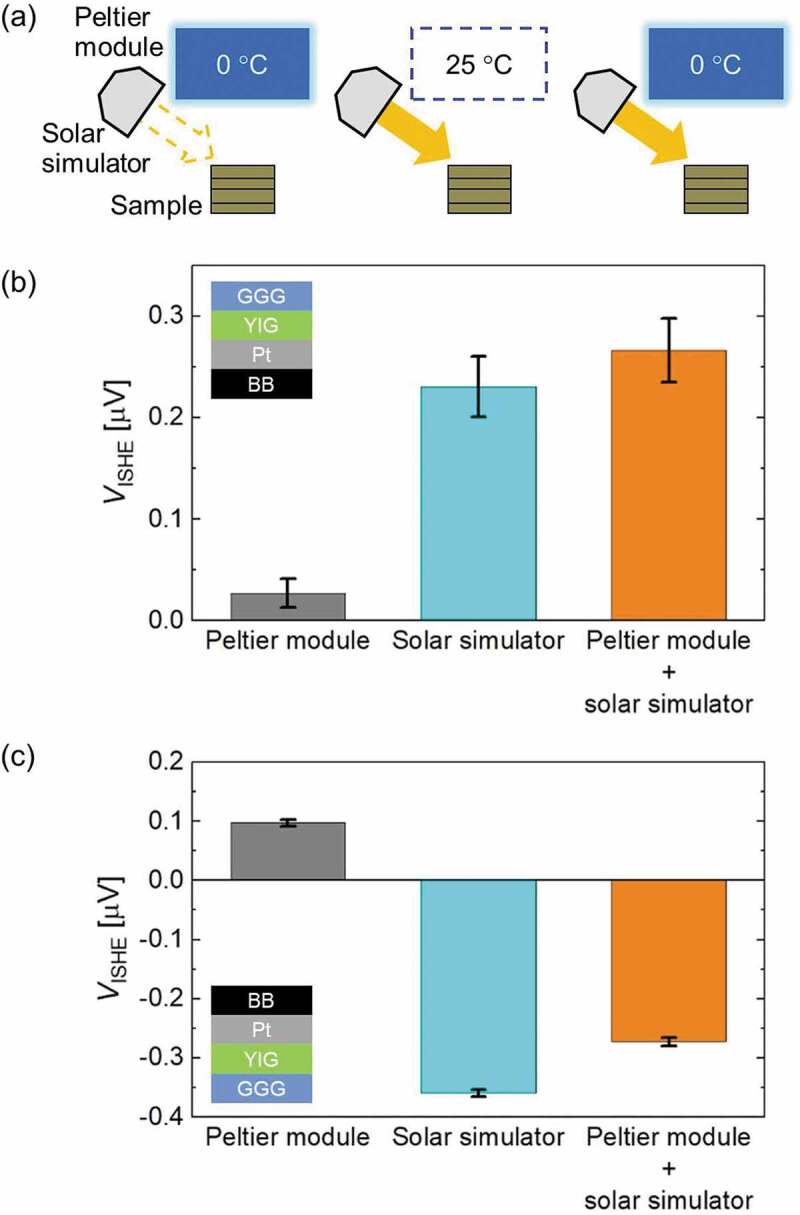
(a) Schematics of the three indoor measurement conditions: radiative cooling by a Peltier module, solar heating by a solar simulator, and radiative cooling and solar heating by a Peltier module and a solar simulator, respectively. The temperatures inside the Peltier module show the surface temperatures of the BB-coated aluminum plate attached to the Peltier module during the indoor measurements. The room temperature was 25°C. Graphs showing the ISHE voltage (*V*_ISHE_) with error bars measured at the three different conditions when the GGG side was facing upward (b), and when the BB side was facing upward (c). Insets show the sample orientations during the measurements

To differentiate the contribution of radiative cooling and solar heating in the *V*_ISHE_ generation, the indoor experiments were carried out using a Peltier module and a solar simulator to mimic outdoor radiative cooling and solar heating, respectively. The *V*_ISHE_ values were recorded at three conditions (only with the Peltier module (*V*_ISHE_(PM)), only with the solar simulator (*V*_ISHE_(SS)), and with both the Peltier module and solar simulator (*V*_ISHE_(PM+SS)) as shown in Figure 4(a). The three values of *V*_ISHE_ when the GGG side was facing upward are plotted in Figure 4(b). All three values were positive and their order was *V*_ISHE_(PM+SS) > *V*_ISHE_(SS) > *V*_ISHE_(PM). These results can be understood if the directions of the temperature gradients caused by the radiative cooling and solar heating are considered. Both radiatively cooling the GGG side and solar heating the BB side caused negative temperature gradients along the *z*-axis. Moreover, since the Peltier module was only cooled to 0°C, the heat flux of the radiative cooling was smaller than the irradiance of the solar simulator, which was also the case with the outdoor experiments. Hence, out of the three conditions, *V*_ISHE_(PM+SS) and *V*_ISHE_(PM) have the largest and smallest values, respectively. Since radiative cooling and solar heating are the major sources of the temperature gradients, the three values naturally satisfy the condition *V*_ISHE_(PM+SS) ~ *V*_ISHE_(PM) + *V*_ISHE_(SS).

The *V*_ISHE_ values at the three indoor conditions when the BB side of the device was facing upward are plotted in Figure 4(c). The figure demonstrates that both *V*_ISHE_(PM+SS) and *V*_ISHE_(SS) are negative, whilst *V*_ISHE_(PM) is positive. In this case, radiative cooling of the BB side caused a negative temperature gradient, however, solar heating of the BB caused a positive temperature gradient. Similar to when the GGG side was facing upward, the heat flux of the radiative cooling was smaller than the irradiance of the solar simulator with opposite signs, such that |*V*_ISHE_(SS)| > |*V*_ISHE_(PM+SS)| > *|V*_ISHE_(PM)|. Note that the relationship of *V*_ISHE_(PM+SS) ~ *V*_ISHE_(PM) + *V*_ISHE_(SS) is also met.

Numerical heat transfer simulations and analytical heat transfer calculations (see Supplementary Note 1) were performed to model the indoor experiments as presented in Figure S3 and Table S2, respectively. Qualitatively, both of the results agree with the indoor experiments. Overall, the indoor experiments and heat transfer analyses verify the outdoor experiments. When the GGG side of the device was facing upward, *V*_ISHE_(day) was larger than *V*_ISHE_(night) because the negative temperature gradient caused by the radiative cooling at night was enhanced by the solar heating in the daytime. When the BB side was facing upward, the sign of *V*_ISHE_(day) was negative and its magnitude was larger than *V*_ISHE_(night). This is because the sign of the temperature gradient by radiative cooling and that by solar heating were opposite and the magnitude of the solar heating temperature gradient was larger.

## 5. Discussion

Our analysis elucidates that by facing the GGG side of the prepared device upwards, it is possible to harvest radiative cooling and solar heating simultaneously for thermoelectric generation by the SSE. As mentioned earlier, the electromagnet can be removed if the remanent magnetization of the ferrimagnetic insulator layer is finite so that there will be no energy consumption to generate *V*_ISHE_. Although previous studies have demonstrated thermoelectric generation either by solar heating or radiative cooling, simultaneous harvesting is the unique characteristic of our device. As our device is based on the SSE, passive cooling and heating without direct contact with solid objects is particularly beneficial to maintain the temperature gradient. Since the SSE devices have a low thickness, heat baths were necessary to maintain temperature gradients. However, the addition of heat baths negatively impacts the favorable characteristics of the SSE devices, such as low thermal resistance and flexibility [6].

Whilst the primary aim of the study was to create a device which exhibits simultaneous harvesting in daytime, our device can also generate voltage even at night by radiative cooling as shown in Figure 3(a). Thus, our device is capable of generating thermoelectric voltage at day or night without any discontinuity, which is distinctly different to photovoltaic cells. Continuous voltage generation may be useful for applications in outdoor energy harvesting to self-power sensors in the IoT society.

The successful demonstration of the simultaneous harvesting device presented in this study may be the focus of future research. In this study, we used ferrimagnetic YIG (paramagnetic Pt) for the heat-to-spin (spin-to-charge) current conversion for the proof-of-concept demonstration. However, in principle, YIG can be replaced with any magnetic materials if they show the SSE and sunlight transparency simultaneously. Pt can be replaced with any conductors including paramagnetic, diamagnetic, ferromagnetic, and antiferromagnetic materials if they show the ISHE. Furthermore, the anomalous Nernst effect (ANE) [38,39] can be used for thermoelectric generation instead of the SSE. The ANE is a transverse thermoelectric effect in a magnetic conductor. Previous studies have demonstrated that devices based on the ANE have similar advantages to SSE devices [40,41]. When designing a thermoelectric device based on the ANE, one needs to ensure sunlight transparency at the top layer as large ANE is often found in opaque metals. Managing the heat conduction inside the device is another important factor to be considered. In the current work, when the GGG side of the device was facing upward, the radiatively cooled GGG top surface was largely separated from the YIG/Pt interface where the *V*_ISHE_ was generated. Optimizing the layer thicknesses increases the temperature gradient around the YIG/Pt interface and resultant SSE-induced thermopower. Other methods which can be utilized to increase the temperature gradient in the device are enhancing the radiative cooling by thermal insulation at the top [29] or tailoring the absorptance of the sunlight absorption layers. Another aspect to be considered is to minimize the solar absorptance of the ferrimagnetic insulator. These presented methods have the potential to improve the thermoelectric generation by the simultaneous harvesting.

## 6. Conclusions

To summarize, we prepared a device that can harvest radiative cooling and solar heating simultaneously to generate thermoelectric voltage by the SSE. The device had a four-layer structure consisting of GGG, YIG, Pt, and BB paint without any complex nanoscale patterning. The GGG and Pt/BB functioned as thermal radiation and sunlight absorbing layers, respectively. The YIG and Pt layers were the essential components for the SSE where YIG (Pt) converts a heat (spin) current into a spin (charge) current. The outdoor and indoor measurements verified that when the GGG side of the device was facing upward, the highest *V*_ISHE_ was obtained when both radiative cooling and solar heating were utilized to generate a temperature gradient perpendicular to the multilayers. In contrast, when the BB side was facing upward, the *V*_ISHE_ from radiative cooling and solar heating was lower than that with only solar heating. We also emphasize that our device can generate *V*_ISHE_ even at night, demonstrating the capability of 24-h electricity generation. Due to the transverse nature of the SSE, the *V*_ISHE_ (power) can increase by laterally elongating the device length (enlarging the device area). Considering the planar and scalable design which does not require any complex fabrications, the device has the potential to be utilized in self-powering off-grid sensors.

## Supplementary Material

Supplemental Material

## References

[1] Uchida K, Takahashi S, Harii K, et al. Observation of the spin Seebeck effect. Nature. 2008;455(7214):778–781. 18843364 10.1038/nature07321

[2] Uchida K, Xiao J, Adachi H, et al. Spin Seebeck insulator. Nat Mater. 2010;9(11):894–897. 20871606 10.1038/nmat2856

[3] Jaworski CM, Yang J, Mack S, et al. Observation of the spin-Seebeck effect in a ferromagnetic semiconductor. Nat Mater. 2010;9(11):898–903. 20871608 10.1038/nmat2860

[4] Uchida K, Adachi H, Ota T, et al. Observation of longitudinal spin-Seebeck effect in magnetic insulators. Appl Phys Lett. 2010;97(17):172505.

[5] Kirihara A, Uchida K, Kajiwara Y, et al. Spin-current-driven thermoelectric coating. Nat Mater. 2012;11(8):686–689. 22706614 10.1038/nmat3360

[6] Kirihara A, Kondo K, Ishida M, et al. Flexible heat-flow sensing sheets based on the longitudinal spin Seebeck effect using one-dimensional spin-current conducting films. Sci Rep. 2016;6(1):23114. 26975208 10.1038/srep23114 PMC4791552

[7] Uchida K, Adachi H, Kikkawa T, et al. Thermoelectric generation based on spin Seebeck effects. Proc IEEE. 2016;104:1499.

[8] Weiler M, Althammer M, Czeschka FD, et al. Local charge and spin Currents in magnetothermal landscapes. Phys Rev Lett. 2012;108(10):106602. 22463435 10.1103/PhysRevLett.108.106602

[9] Agrawal M, Vasyuchka VI, Serga AA, et al. Role of bulk-magnon transport in the temporal evolution of the longitudinal spin-Seebeck effect. Phys Rev B. 2014;89(22):224414.

[10] Ishii S, Uchida K, Dao TD, et al. Wavelength-selective spin-current generator using infrared plasmonic metamaterials. APL Photonics. 2017;2(10):106103.

[11] Telkes M. Solar thermoelectric generators. J Appl Phys. 1954;25(6):765–777.

[12] Hasebe M, Kamikawa Y, Meiarashi S, editors. Thermoelectric generators using solar thermal energy in heated road pavement. 2006 25th International Conference on Thermoelectrics; 2002 Aug 6–10; Vienna, Austria.

[13] Kraemer D, Poudel B, Feng H-P, et al. High-performance flat-panel solar thermoelectric generators with high thermal concentration. Nat Mater. 2011;10(7):532–538. 21532584 10.1038/nmat3013

[14] Yang D, Yin H. Energy conversion efficiency of a novel hybrid solar system for photovoltaic, thermoelectric, and heat utilization. IEEE Trans Energy Convers. 2011;26(2):662–670.

[15] Huen P, Daoud WA. Advances in hybrid solar photovoltaic and thermoelectric generators. Renew Sust Energ Rev. 2017;72:1295–1302.

[16] Yamada A, inventorThermoelectric conversion system (Japanese patent). Japan patent JPB 002716861. 1997.

[17] Raman AP, Li W, Fan S. Generating light from darkness. Joule. 2019;3:1–8.

[18] Catalanotti S, Cuomo V, Piro G, et al. The radiative cooling of selective surfaces. Sol Energy. 1975;17(2):83–89.

[19] Nilsson TMJ, Niklasson GA, Granqvist CG. A solar reflecting material for radiative cooling applications: ZnS pigmented polyethylene. Sol Energy Mater Sol Cells. 1992;28(2):175–193.

[20] Raman AP, Anoma MA, Zhu L, et al. Passive radiative cooling below ambient air temperature under direct sunlight. Nature. 2014;515:540. 25428501 10.1038/nature13883

[21] Sun X, Sun Y, Zhou Z, et al. Radiative sky cooling: fundamental physics, materials, structures, and applications. Nanophotonics. 2017;6(5):997–1015.

[22] Zeyghami M, Goswami DY, Stefanakos E. A review of clear sky radiative cooling developments and applications in renewable power systems and passive building cooling. Sol Energy Mater Sol Cells. 2018;178:115–128.

[23] Zhao D, Aili A, Zhai Y, et al. Radiative sky cooling: fundamental principles, materials, and applications. Appl Phys Rev. 2019;6(2):021306.

[24] Hsu P-C, Song AY, Catrysse PB, et al. Radiative human body cooling by nanoporous polyethylene textile. Science. 2016;353(6303):1019–1023. 27701110 10.1126/science.aaf5471

[25] Kou J, Jurado Z, Chen Z, et al. Daytime radiative cooling using near-black infrared emitters. ACS Photonics. 2017;4(3):626–630.

[26] Zhai Y, Ma Y, David SN, et al. Scalable-manufactured randomized glass-polymer hybrid metamaterial for daytime radiative cooling. Science. 2017;355(6329):1062–1066. 28183998 10.1126/science.aai7899

[27] Mandal J, Fu Y, Overvig AC, et al. Hierarchically porous polymer coatings for highly efficient passive daytime radiative cooling. Science. 2018;362(6412):315–319. 30262632 10.1126/science.aat9513

[28] Bhatia B, Leroy A, Shen Y, et al. Passive directional sub-ambient daytime radiative cooling. Nat Commun. 2018;9(1):5001. 30479326 10.1038/s41467-018-07293-9 PMC6258698

[29] Leroy A, Bhatia B, Kelsall CC, et al. High-performance subambient radiative cooling enabled by optically selective and thermally insulating polyethylene aerogel. Sci Adv. 2019;5(10):eaat9480. 31692957 10.1126/sciadv.aat9480 PMC6821464

[30] Li T, Zhai Y, He S, et al. A radiative cooling structural material. Science. 2019;364(6442):760–763. 31123132 10.1126/science.aau9101

[31] Luo H, Li Q, Du K, et al. An ultra-thin colored textile with simultaneous solar and passive heating abilities. Nano Energy. 2019;65:103998.

[32] Heo S-Y, Lee GJ, Kim DH, et al. A *Janus* emitter for passive heat release from enclosures. Sci Adv. 2020;6(36):eabb1906. 32917610 10.1126/sciadv.abb1906 PMC7473666

[33] Azevedo A, Leão LHV, Rodriguez-Suarez RL, et al. dc effect in ferromagnetic resonance: evidence of the spin-pumping effect? J Appl Phys. 2005;97(10):10C715.

[34] Saitoh E, Ueda M, Miyajima H, et al. Conversion of spin current into charge current at room temperature: inverse spin-Hall effect. Appl Phys Lett. 2006;88(18):182509.

[35] Valenzuela SO, Tinkham M. Direct electronic measurement of the spin Hall effect. Nature. 2006;442(7099):176–179. 16838016 10.1038/nature04937

[36] Wood DL, Nassau K. Optical properties of gadolinium gallium garnet. Appl Opt. 1990;29(25):3704–3707. 20567472 10.1364/AO.29.003704

[37] Rakić AD, Djurišić AB, Elazar JM, et al. Optical properties of metallic films for vertical-cavity optoelectronic devices. Appl Opt. 1998;37(22):5271–5283. 18286006 10.1364/ao.37.005271

[38] Lee W-L, Watauchi S, Miller VL, et al. Anomalous Hall heat current and Nernst effect in the CuCr_2_Se_4−x_Br_x_ ferromagnet. Phys Rev Lett. 2004;93(22):226601. 15601108 10.1103/PhysRevLett.93.226601

[39] Miyasato T, Abe N, Fujii T, et al. Crossover behavior of the anomalous Hall effect and anomalous Nernst effect in itinerant ferromagnets. Phys Rev Lett. 2007;99(8):086602. 17930968 10.1103/PhysRevLett.99.086602

[40] Sakuraba Y. Potential of thermoelectric power generation using anomalous Nernst effect in magnetic materials. Scr Mater. 2016;111:29–32.

[41] Uchida K, Zhou W, Sakuraba Y. Transverse thermoelectric generation using magnetic materials. Appl Phys Lett. 2021;118(14):140504.

