# Supplementary material for: Simultaneous harvesting of radiative cooling and solar heating for transverse thermoelectric generation
Source: Sci Technol Adv Mater. 2021 Jun 29;22(1):441–8. doi: 10.1080/14686996.2021.1920820 (PMC8245095; doi:10.1080/14686996.2021.1920820)
Supplement: Supplemental Material [file TSTA_A_1920820_SM8088.pdf]

Supplementary Materials

Simultaneous harvesting of radiative cooling and solar heating for transverse thermoelectric generation

Satoshi Ishii,[a,b,c*] Asuka Miura,[d,‡] Tadaaki Nagao,[a,e] and Ken-ichi Uchida[b,d,f,g*]

[a] *International Center for Materials Nanoarchitectonics (MANA), National Institute for Materials Science (NIMS), Tsukuba, Ibaraki 305-0044, Japan*

[b] *Faculty of Pure and Applied Physics, University of Tsukuba, Tsukuba, Ibaraki 305-8577, Japan*

[c] *PRESTO, Japan Science and Technology Agency, Saitama 332-0012, Japan*

[d] *Research Center for Magnetic and Spintronic Materials, National Institute for Materials Science (NIMS), Tsukuba, Ibaraki 305-0047, Japan*

[e] *Department of Condensed Matter Physics, Graduate School of Science, Hokkaido University, Sapporo, Hokkaido 060-0810, Japan*

[f] *Institute for Materials Research, Tohoku University, Sendai, Miyagi 980-8577, Japan*

[g] *Center for Spintronics Research Network, Tohoku University, Sendai, Miyagi 980-8577, Japan*

[‡] *Present address: Integrated Research for Energy and Environment Advanced Technology, Kyushu Institute of Technology, Kitakyushu, Fukuoka 804-8550, Japan*

*E-mail: sishii@nims.go.jp; UCHIDA.Kenichi@nims.go.jp

Supplementary Note 1. Heat transfer analysis.

A heat transfer analysis was performed in a simplified 2D geometry to model the indoor experiment. The net emission power P_{net} at the top and bottom of the sample when the GGG side is facing upward are expressed as

$$P_{\text{net}}^{\text{top}}(T_{\text{top}}, T_{\text{bottom}}, T_{\text{amb}}) = P_{\text{rad}}(T_{\text{top}}) + P_{\text{conv}}^{\text{top}}(T_{\text{top}}, T_{\text{amb}}) - P_{\text{cond}}^{\text{sample}}(T_{\text{top}}, T_{\text{bottom}}) \quad \text{(S1)}$$

$$P_{\text{net}}^{\text{bottom}}(T_{\text{top}}, T_{\text{bottom}}, T_{\text{amb}}) = P_{\text{cond}}^{\text{sample}}(T_{\text{top}}, T_{\text{bottom}}) + P_{\text{conv}}^{\text{bottom}}(T_{\text{top}}, T_{\text{amb}}) + P_{\text{cond}}^{\text{support}}(T_{\text{bottom}}, T_{\text{amb}}) - P_{\text{sun}}, \quad \text{(S2)}$$

where P_{rad}, P_{sun}, P_{conv}, and P_{cond} are the radiated power from the sample surface at the top sample temperature T_{top}, the absorbed power by the incident sunlight, the convective heat loss, and conductive heat loss inside the sample. Other temperatures, T_{bottm} and T_{amb}, represent the bottom sample temperature and ambient temperature, respectively. The conductive heat losses were separated into the conductions inside the sample ($P_{\text{cond}}^{\text{sample}}$) and conductions in the sample support ($P_{\text{cond}}^{\text{support}}$).

When calculating P_{rad}, and P_{sun}, the spectroscopic emissivity (from the UV to MIR) of the sample was considered, and the atmospheric transmittance was assumed to be 100 % because the distance between the sample and Peltier module was ~25 mm which was very close. Since the Peltier module was cooled only to 273 K, the radiation from the Peltier module was taken into account in the calculation of P_{rad}. The thermal properties were taken from the references [1,2]. The room temperature of the lab, which was 25 °C, was used as T_{amb}. The convective heat transfer coefficient was set to 10 W/m^2/K and thermal resistances in the conductive heat transfer were estimated based on the material properties. By setting P_{net} = 0 in Eqs. (S1) and (S2), T_{top}, and T_{bottom} were solve iteratively.

Similarly, the net emission power P_{net} at the top and bottom of the sample when the BB side is facing upward are expressed as

$$P_{\text{net}}^{\text{top}}(T_{\text{top}}, T_{\text{bottom}}, T_{\text{amb}}) = P_{\text{rad}}(T_{\text{top}}) + P_{\text{conv}}^{\text{top}}(T_{\text{top}}, T_{\text{amb}}) - P_{\text{cond}}^{\text{sample}}(T_{\text{top}}, T_{\text{bottom}}) - P_{\text{sun}} \quad \text{(S3)}$$

$$P_{\text{net}}^{\text{bottom}}(T_{\text{top}}, T_{\text{bottom}}, T_{\text{amb}}) = P_{\text{cond}}^{\text{sample}}(T_{\text{top}}, T_{\text{bottom}}) + P_{\text{conv}}^{\text{bottom}}(T_{\text{top}}, T_{\text{amb}}) + P_{\text{cond}}^{\text{support}}(T_{\text{bottom}}, T_{\text{amb}}). \quad \text{(S4)}$$

Equations (S3) and (S4) were also iteratively solved for T_{top} and T_{bottm} by setting P_{net} = 0.

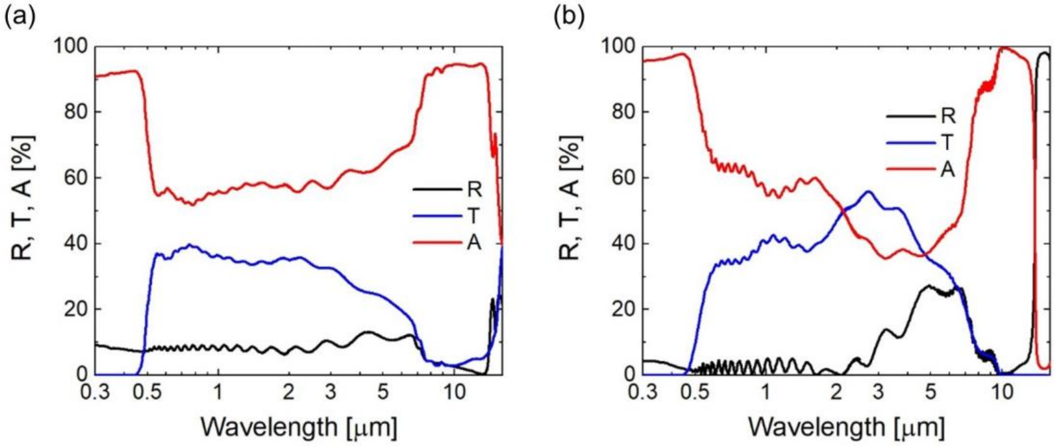

Fig. S1. Optical properties of the GGG/YIG/Pt sample. Measured (a) and calculated (b) reflectance (R), transmittance (T), and absorptance (A) of the GGG/YIG/Pt sample without the BB layer. In the measurement and calculation, the incident light was from the GGG side.

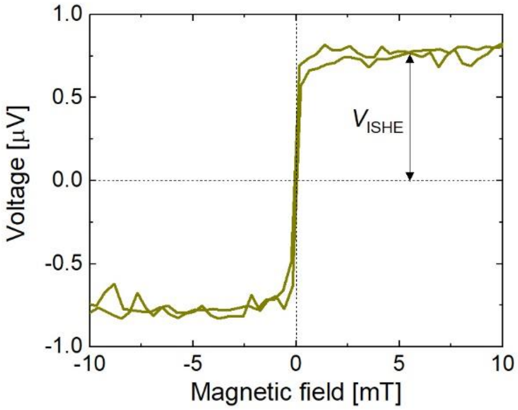

Fig. S2. H-V curve. Magnetic field dependence of the voltage measured outdoor at 9 am on 5th May, 2019. Three measurements were averaged and the offset due to the field-independent thermoelectric voltage was subtracted. The definition of V_{ISHE} is shown in the figure.

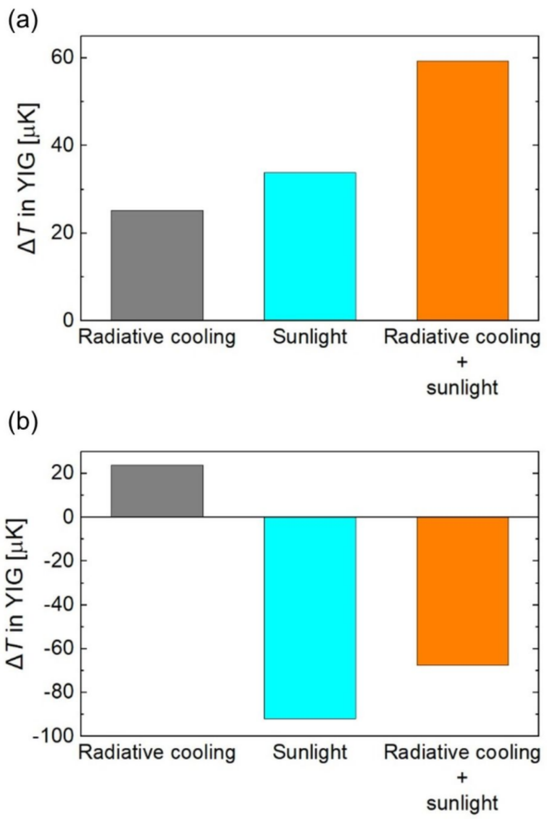

Fig. S3. Numerical simulations for the indoor experiments. Numerically simulated temperature differences (ΔT) between top and bottom of the YIG film when the GGG side was facing upward (a) and when the BB side was facing upward (b). The heat transfer simulations were based on the finite element method (COMSOL Multiphysics). In the simulations, the thermal properties of the materials which consisted the sample except the BB were taken from ref. [1], and that of the BB which was assumed to be iron oxide was taken from ref. [2]. The simulated geometry referred to the actual sample which included the sample supports. The support had a fixed temperature as a boundary condition. The radiative cooling was modeled by setting the ambient temperature to the Peltier module temperature in the radiative heat transfer option. The solar heating by sunlight was modeled as an incoming heat flux. The simulations were performed by facing the GGG side upward and the BB side upward.

Table S1. Summary of the outdoor experiments. Weather conditions at the time of the measurements and the measured ISHE voltage (V_{ISHE}) where either the GGG side or BB side was facing up. The temperature and humidity data were the recorded at the meteorological observatory in Tsukuba, Japan and the data were taken from the Japan Meteorological Agency website. The distance between the meteorological observatory and measured point is around 1360 m. The solar irradiance was measured within 30 cm from the sample.

Top side	Time & Date	Weather	Temperature [°C]	Humidity [%]	Solar irradiance [mW/cm^2]	V_{ISHE} [μV]
GGG	10 pm, 4th May	Sunny	13.7	91	0	0.04
GGG	9 am, 5th May	Sunny	19.6	60	61	0.77
GGG	4 pm, 4th May	Cloudy	20.3	58	8	0.12
BB	8 pm, 16th May	Sunny	18.5	73	0	0.15
BB	9 am, 16th May	Sunny	21.0	73	65	-0.83
BB	9 pm, 14th May	Cloudy	18.5	78	6	-0.06

Table S2. Summary of the analytical heat transfer analysis. Analytically estimated temperature differences (ΔT) between the top (T_{top}) and bottom (T_{bottom}) of the sample in the indoor experiment. The analytical heat transfer calculation was based on the method described in Note 1.

Top side	Condition	ΔT [K]
GGG	Cooling	0.0041
	Sunlight	0.0087
	Cooling + sunlight	0.0146
BB	Cooling	0.0055
	Sunlight	-0.0265
	Cooling + sunlight	-0.0179

References

1. Schreier M, Kamra A, Weiler M, et al. Magnon, phonon, and electron temperature profiles and the spin Seebeck effect in magnetic insulator/normal metal hybrid structures. Phys Rev B. 2013;88(9):094410.
2. Takeda M, Onishi T, Nakakubo S, et al. Physical properties of iron-oxide scales on Si-containing steels at high temperature. Mater Trans. 2009;50(9):2242-2246.
